# Melatonin Prevents Cisplatin-Induced Cyto-Histopathological Damage in the Bone Marrow and Inner Ear

**DOI:** 10.1055/s-0045-1809645

**Published:** 2025-09-10

**Authors:** Juliana Gusmão de Araujo, Lucieny Silva Martins Serra, Lucas Lauand, Selma Aparecida Souza Kückelhaus, André Luiz Lopes Sampaio

**Affiliations:** 1 Teaching and Research Laboratory in Otorhinolaryngology, Faculty of Medicine, Universidade de Brasília, Brasília, DF, Brazil

**Keywords:** ototoxicity, cisplatin, melatonin, genotoxicity

## Abstract

**Introduction:**

Cisplatin is an effective chemotherapeutic drug. Its side effects, ototoxicity and genotoxicity, limit widespread application. Melatonin could reduce these toxic effects due to its antioxidant activity.

**Objective:**

To determine the effect of melatonin against cisplatin-induced ototoxicity and genotoxicity in rats.

**Methods:**

To assess ototoxicity, 33 rats were randomly divided into: group 1 (saline), group 2 (melatonin), group 3 (cisplatin + saline), and group 4 (cisplatin + melatonin). Groups 3 and 4 received a single dose of 10 mg/kg of cisplatin. Groups 2 and 4 received daily doses of 1 mg/kg of melatonin. The number of viable neurons and their average diameter in the spiral and vestibular ganglia were analyzed. In the stria vascularis and spiral ligament, the number of viable cells was evaluated. To assess genotoxicity, 12 animals were randomly divided into: group A (saline), group B (cisplatin + saline), and group C (cisplatin + melatonin). The rats in groups B and C received a single dose of 10 mg/kg of cisplatin. Group C received a single dose of 1 mg/kg of melatonin.

**Results:**

The micronucleus count and percentage of polychromatic erythrocytes in the bone marrow of rat femurs were analyzed. The animals in group 3 presented a greater loss of cells than the animals in other groups regarding all cochlear structures evaluated. Furthermore, the diameters of neurons were smaller in the animals in group 3. Melatonin-treated rats presented a lower micronucleus count and a higher number of polychromatic erythrocytes than animals treated with cisplatin alone.

**Conclusion:**

Melatonin reduces cisplatin-induced cyto-histopathological damage in the bone marrow and inner ear; therefore, it could be used as a tumor adjuvant treatment.

## Introduction

Cisplatin is a highly effective and widely employed antineoplastic agent. It was approved by the United States Food and Drug Administration (FDA) in 1978, initially for ovarian, testicular, and bladder tumors. This drug transformed the prognosis of previously lethal malignancies, including testicular cancer, curing up to 80% of the cases. To date, it is used to treat various solid tumors in adults and children. 1

The cytotoxic effect of cisplatin is attributed to its DNA binding with the formation of adducts, with intra- and interchain bonds that lead to structural changes in DNA. Consequently, transcription and replication are inhibited, resulting in the induction of apoptosis of tumor cells. Furthermore, cisplatin produces free radicals, reduces antioxidant defense mechanisms, and activates the caspase cascade. However, these effects of cisplatin are not restricted to cancer cells, with diverse tissues and organs susceptible to its action. 2 3

Thus, despite its widespread and routine use, associated side effects impact the patient's quality of life. Even though the adoption of certain therapeutic measures can minimize the nephrotoxic and gastrointestinal effects, ototoxicity and genotoxicity still lack proven strategies for prevention. 1

The prevalence of cisplatin-induced ototoxicity is highly variable due to the different population characteristics investigated, including age, route of administration, and cumulative dose, ranging from 20 to 90%. 4 5

Cisplatin can cause cochlear damage at high acute and high cumulative doses. Reportedly, on reaching the organ of Corti, cisplatin initiates its deleterious action on supporting cells, followed by the outer hair cells, predominantly in the middle and basal turn, and later in the stria vascularis and cells of the spiral ganglion. 6 7 8 9

Ototoxicity can be manifested as hearing loss, tinnitus, or vertigo. The hearing loss is generally bilateral, sensorineural, mainly at high frequencies, dose-dependent, progressive, and irreversible. 10 Particularly in children, the consequences of hearing loss can be catastrophic due to the impact on language acquisition and cognitive development. 11

In terms of genotoxic activity, cisplatin inhibits cell proliferation through several different mechanisms, including direct DNA damage, changes in DNA metabolism, as well as through the mitotic process. 12 Successful cancer treatments eliminate tumor cells, but they can also induce genomic changes in healthy tissues. 13 The mutagenicity of cisplatin is associated with sister-chromatid exchange and chromosomal aberrations, especially in bone marrow cells. 12

It has been reported 14 15 that patients who survive for long periods after cancer treatments have a higher incidence of secondary tumors in different organs due to mutations induced by chemotherapy. Furthermore, treatment-induced mutations in surviving cancer cells increase the genetic heterogeneity of the tumor and may contribute to the development of resistance. 14

Although the ototoxic and genotoxic effects of the entire chemotherapeutic subgroup to which cisplatin belong are already well described, their exact mechanism needs to be comprehensively elucidated. The most accepted hypothesis is the production of free radicals and the reduction of antioxidant defense mechanisms, with activation of the caspase cascade, resulting in cellular apoptosis. 16 Another accepted mechanism is the altered production of proteins of the *BCL2* gene family, which is responsible for the control of cellular apoptosis. 17

Melatonin is an endogenously produced substance best known for its ability to synchronize circadian rhythm. 18 Reportedly, melatonin stimulates the synthesis of antioxidant enzymes and protects them from oxidative damage, which sometimes impairs their proper functioning. 19 20

Therefore, based on the well-established antioxidant effect of melatonin, it is expected that its use in the present study can establish new perspectives for the prevention of ototoxicity and genotoxicity during chemotherapeutic treatment with cisplatin, with potential future biomedical and biotechnological applications.

## Methods

The current is a prospective and intervention study approved by the institutional Ethics Committee on Animal Use. We used 45 female Wistar rats, 6 to 8 weeks of age, and weighing between 150 g and 250 g. The animals were housed at room temperature (25 ± 3° C), with a 12-hour light/dark cycle, and access to balanced food and drinking water ad libitum.

### Assessment of Ototoxicity

In total, 33 animals were anesthetized with ketamine hydrochloride (75 mg/kg) and xylazine (5 mg/kg) for us to perform otoscopy and obtain distortion product otoacoustic emissions (DPOAE) with the ILO 292 equipment (Otodynamics Ltd.). An acoustic-insulated box was devised for this test. The stimulus consisted of 2 pure tones (F1 and F2; F1/F2 ratio = 1.22) at 70 dB SPL. One thousand acquisitions were analyzed. The resulting otoacoustic emissions were rated at 2.8 kHz, 4.0 kHz, 6.0 kHz, and 8.0 kHz. 21

Animals with any alterations in the middle and/or outer ear were excluded, along with animals with absent DPOAE at any of the frequencies before drug administration. Then, the animals were randomly assigned to four groups: **group 1** (5 animals) – saline; **group 2** (5 animals) – melatonin (1 mg/kg); **group 3** (12 animals) – cisplastin (10 mg/kg) + saline; and **group 4** (11 animals) – cisplatin (10 mg/kg) + melatonin (1 mg/kg).

Melatonin was weighed daily on a precision scale and diluted as recommended by the manufacturer. The applications, performed intraperitoneally, were started on the first day of the study (D1) and maintained daily, until the eighth day (D8) in groups 2 and 4. Cisplatin was stored in a dark glass container at room temperature. In groups 3 and 4, cisplatin was administered as a single dose of 10 mg/kg on the fourth day of the experiment (D4).

On D8, the animals were sacrificed and perfused with 20 mL of 10% formaldehyde solution via a catheter inserted into the left ventricle and right atrium opening. The head was removed and placed in the same fixative solution for at least 12 hours. Incomplete descaling was performed for 24 hours in an ethylenediaminetetraacetic acid (EDTA) solution (0.78 mg/95 mL of running water) plus 5 mL of nitric acid purissima analysis (p.a.). Next, the head was washed under running water for 2 hours, followed by opening of the calvaria in the median region and removal of the entire brain with the aid of thick-tipped forceps. The right acoustic canal was identified with a string or suture thread, and the anterior region of the head was separated. Next, descaling was completed, followed by dehydration in alcohol, diaphanization in xylol, and impregnation in paraffin (60° C). Finally, 6-μm thick sections were obtained in the horizontal plane and stained with hematoxylin and eosin.

The images of histological sections (5 µm) were captured on the Aperio ScanScope® equipment (Aperio Technologies Inc., Vista, CA, USA) and the morphological analyses were performed by an observer in the program ImageScope version 11.2.0.780 (Aperio Technologies Inc).

The spiral ganglion, vestibular ganglion, stria vascularis, and spiral ligament were evaluated. From each animal, three different slides in the midmodiolar plane were selected, with no significant artifacts such as tissue retraction, folds, or membrane disruption. The number of viable cells per pre-established area and the mean diameter of the neurons were analyzed, obtained as the average between the largest and smallest diameters of ten cells randomly chosen in each histological section analyzed.

### Assessment of Genotoxicity

To assess genotoxicity, 12 animals were randomly divided into 3 groups: **group A** (4 animals) – saline; **group B** (4 animals) – cisplatin (10 mg/kg) + saline; and **group C** (4 animals) – cisplatin (10 mg/kg) + melatonin (1 mg/kg).

The drugs were intraperitoneally administered in a single dose. After 48 hours, the animals were sacrificed and the bone marrow was extracted by washing the femurs with saline, followed by gentle homogenization. All specimens were fixed in a 10% formaldehyde solution and processed to obtain 5-μm thick histological sections. After staining, the histological sections were photographed at a magnification of 1,000× using a microscope (Carl Zeiss Microscopy, LLC), and then analyzed by a single observer blinded to group distribution.

The quantification of micronuclei (MNs), polychromatic erythrocytes (PCEs), and normochromatic erythrocytes (NCEs) was performed by analyzing 1,000 erythrocytes per animal. The results are expressed by the frequency of MNs in the sample and by the percentage of PCEs as follows: [PCE/(PCE + NCE)] x 100.

### Analytical Procedures

Normality was assessed using the Kolmogorov-Smirnov and Shapiro-Wilk tests. Then, analysis of variance (ANOVA) was performed, followed by the student-Newman-Keuls method (parametric data) or Kruskal-Wallis method for multiple comparisons, and by the Dunn method (nonparametric data). Values of *p* < 0.05 were considered statistically significant. All analyses and graphical representations were performed using the Prism 5 software package (GraphPad Software, Inc.).

## Results

### Ototoxicity

#### Spiral Ganglion

**Fig. 1:**
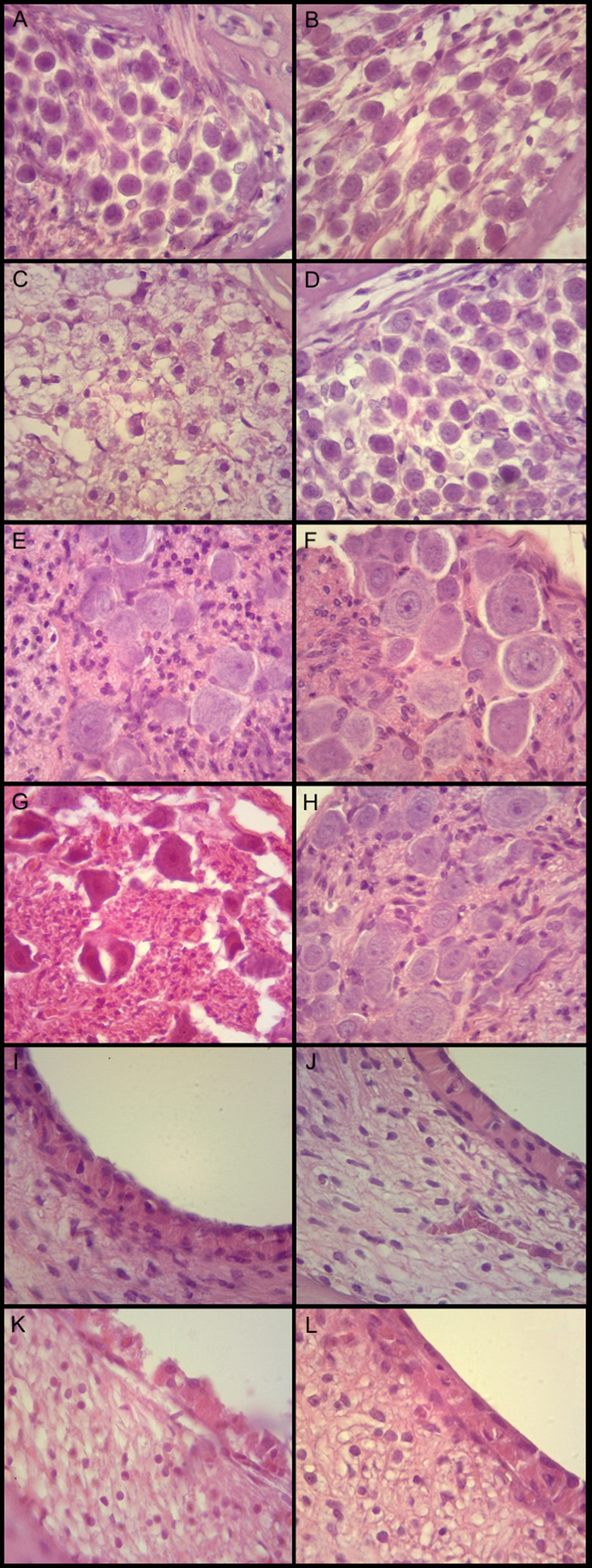
Photomicrographs of the spiral ganglion.

**Fig. 2:**
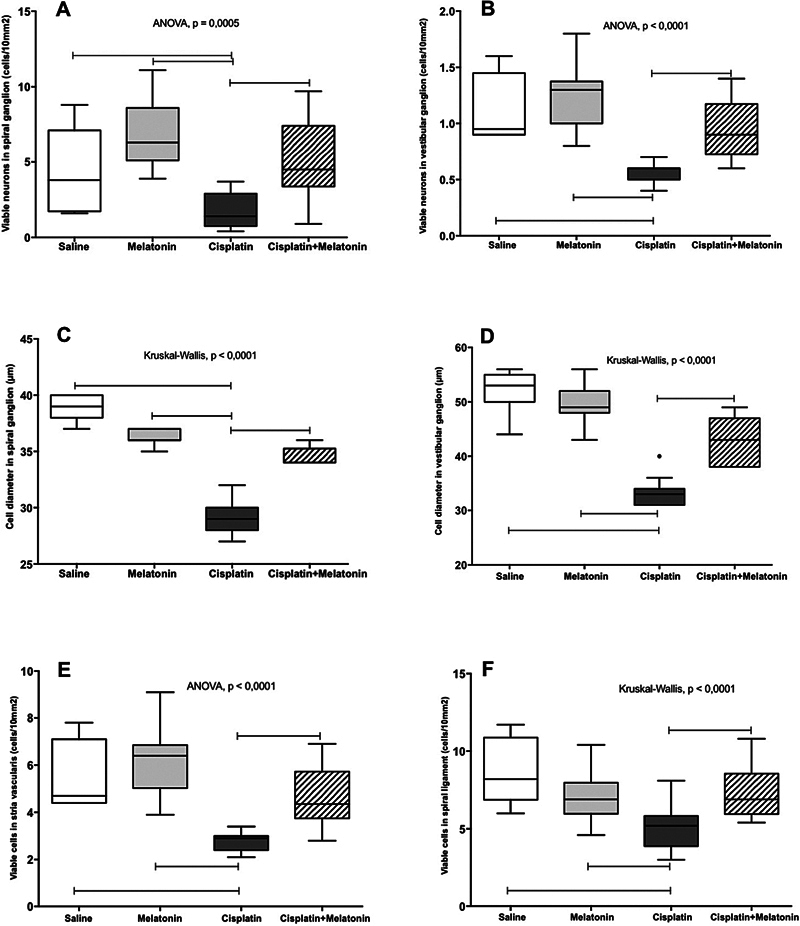
Evaluation of the spiral ganglion ( **A, C** ), vestibular ganglion ( **B, D** ), stria vascularis ( **E** ) and spiral ligment ( **F** ).

In terms of cell density, a significant difference was observed regarding group 3 (1.7 cells/mm ^2^ ) and groups 1 (4.4 cells/mm ^2^ ), 2 (6.8 cells/mm ^2^ ), and 4 (5.4 cells/mm ^2^ ) ( *p* -value = 0.0004) ( Fig. 1A–D ; Fig. 2A ).

Regarding the mean cell diameter of the neurons, a statistical difference ( *p* -value < 0.0001) was observed regarding group 3 (29 µm) and groups 1, 2, and 4, which presented values of 39 µm, 36 µm, and 34 µm respectively ( Fig. 1A–D ; Fig. 2C )

#### Vestibular Ganglion

For cell density, a significant difference was observed regarding group 3 (0.5 cells/mm ^2^ ) and groups 1 (1.2 cells/mm ^2^ ), 2 (1.2 cells/mm ^2^ ), and 4 (0.9 cells/mm ^2^ ) ( *p* -value = 0.0001) ( Fig. 1E–H ; Fig. 2B ).

Regarding the mean cell diameter of neurons, a statistical difference ( *p* -value < 0.0001) was observed regarding group 3 (33 µm) and groups 1, 2, and 4, which presented values of 52 µm, 49 µm, and 43 µm respectively ( Fig. 1E–H ; Fig. 2D ).

#### Stria Vascularis

In terms of cell density, a significant difference was observed regarding group 3 (2.7 cells/mm ^2^ ) and groups 1 (5.4 cells/mm ^2^ ), 2 (6.2 cells/mm ^2^ ), and 4 (4.6 cells/mm ^2^ ) ( *p* -value = 0.0001) ( Fig. 1I–L ; Fig. 2E ).

#### Spiral Ligament

Regarding cell density, a significant difference was observed regarding group 3 (5.2 cells/mm ^2^ ) and groups 1 (8.2 cells/mm ^2^ ), 2 (6.7 cells/mm ^2^ ), and 4 (6.9 cells/mm ^2^ ) ( *p* -value = 0.0001) ( Fig. 1I–L ; Fig. 2F ).

#### Genotoxicity

**Fig. 3:**
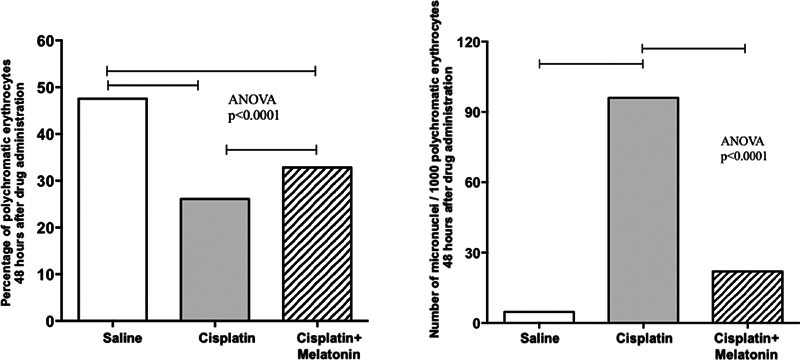
Percentage of polychromatic erythrocytes (PCEs) and number of micronuclei (MNs) of rat bone marrow erythrocytes after 48 hours of drug administration.

The mean percentage of PCEs decreased in groups B (26.1 ± 1.1%) and C (32.8 ± 1.7%) when compared with group A (47.6 ± 3.1%) ( *p* < 0.05). A statistical difference was observed between groups B and C ( *p* < 0.05) ( Fig. 3 )

The number of MNs in group A (4.8 ± 0.9 MNs/1,000 PCEs) was considerably lower than in groups B (22.0 ± 5.2 MN/1,000 PCEs) and C (96.0 ± 19.6 MN/1,000 PCEs) ( *p* < 0.05). No statistically significant difference was observed between groups A and C ( Fig. 3 ).

## Discussion

Prevention of cisplatin side effects remains a major challenge. Ototoxicity and genotoxicity, unlike other adverse effects, have no established measures proven to minimize them.

In terms of ototoxicity, the results obtained in the present study showed that the single dose of 10 mg/kg of cisplatin was sufficient to cause cyto-histological changes in the spiral ganglion, vestibular ganglion, stria vascularis, and spiral ligament.

For genotoxic effects, a reduction in the percentage of PCEs and an increase in the number of MNs was observed in groups treated with cisplatin, possibly resulting from the death of stem cells induced by cytotoxic damage. Furthermore, the protective action of melatonin was observed in the treated group.

The protective effects of other antioxidant agents concurrently administered with cisplatin have been previously suggested; nevertheless, none of these agents are routinely employed. 22 23 In addition to analyzing the systemic toxicity of the proposed drug, it is essential to assess its degree of interaction with other drugs in the chemotherapy protocol and to identify any possible negative effects on cancer treatment.

Melatonin has been previously evaluated in several animal species and at different doses, with no major side effects reported even at high doses, 24 25 26 as observed in the present investigation. The concomitant administration of melatonin with chemotherapeutic treatments reduces adverse events that increase mortality, including thrombocytopenia, neurotoxicity, and cardiotoxicity. 27 Several studies have previously demonstrated the benefit of using melatonin during chemotherapy for solid tumors. 28 29 30 31 32 Furthermore, studies have indicated the benefit of using melatonin in neuroblastoma, gliomas, lymphoproliferative diseases, as well as pancreatic and colorectal tumors. 33 34 35 36 37 In patients with breast cancer, the coadministration of melatonin with tamoxifen results in better treatment response, longer one-year survival, and reduced levels of anxiety and depression. 38

In 2012, Wang et al. 39 published a systematic review and meta-analysis of randomized clinical trials on the efficacy and safety of melatonin administration combined with chemotherapy or radiotherapy in solid tumors. In most studies, melatonin therapy reportedly resulted in higher rates of tumor remission, better one-year survival, and fewer side effects related to radiotherapy and chemotherapy, including thrombocytopenia, neurotoxicity, and fatigue. 39

Specifically analyzing the adverse effects of cisplatin, melatonin can protect against kidney damage. 40 41 42 In terms of ototoxicity, there is evidence suggesting that melatonin affords protection against gentamicin- and tobramycin-induced hearing damage without interfering with antibiotic actions. 43 44

Reportedly, ototoxic effects can be observed within 72 hours of cisplatin administration. 45 In the present study, the analysis was performed 96 hours after the cisplatin treatment, which was sufficient to demonstrate the lesions resulting from cisplatin administration. After 96 hours, mortality was highly increased in rats exposed to chemotherapy; 46 therefore, the long-term protective effects of melatonin should be evaluated preferably using another animal model.

In addition to the changes classically observed in the organ of Corti, such as loss of internal and external hair cells, cisplatin is known to cause extensive loss of cochlear cytoarchitecture, changes in the stria vascularis, and rarefaction of neurons in the spiral ganglion. 47 48 49

Analyzing the stria vascularis and the spiral ligament, we observed a statistically significant difference regarding group 3 (cisplatin) and all other groups, indicating that the administration of melatonin affords protection against the damage induced by cisplatin. Cisplatin caused an intense change in the cytoarchitecture of the stria vascularis, with marked cell loss. These changes were reduced with the concomitant use of melatonin.

In stria vascularis, reversible histological lesions have been reported in the available literature. 49 However, as the present study involved a short-term experiment, we could not define whether stria vascularis damages would be sustained in the long term.

Cisplatin can affect peripheral sensory nerves and induce neuropathies. 49 In the current study, melatonin minimized neuronal death and cisplatin-induced ganglion cell degeneration. The toxic effects of cisplatin on the spiral ganglion are well-established; however, lesions to the vestibular ganglion are not typically described. In the present study, the cytoarchitecture of the spiral and vestibular ganglia was damaged, with evident rarefaction and reduced diameter of neurons.

Furthermore, studies have revealed considerably variable vestibulotoxicities associated with cisplatin. 50 51 Although impaired posture and vestibulo-ocular reflex have been noted in animal investigations, histopathological analyses usually show preservation of the neuroepithelium of the posterior labyrinth. 6 The reduced number and diameter of neurons in the vestibular ganglion observed in the current study could partly justify the clinical symptoms described.

In terms of genotoxicity, the genomic changes caused by cisplatin have been well-established. Damage to DNA can result not only in cell death but also in mutations in both somatic and germ cells. Due to its carcinogenic potential, cisplatin can induce the development of secondary tumors, particularly in pediatric patients. 14 Proliferative cells, such as those in the bone marrow, are more susceptible, and an increased incidence of leukemia has been reported. 52 Although the mechanism underlying cisplatin resistance is complex, the genotoxic damage induced by the drug increases the genetic heterogeneity of the tumor, which may contribute to the development of chemotherapy resistance. 14

In the groups treated with cisplatin, the percentage of PCEs decreased when compared with the negative control group. Importantly, a higher percentage of PCEs was observed in the cisplatin + melatonin group (group C) compared to the cisplatin group (group B). These data are indicative of cell toxicity, with inhibition of cell division, as well as cell death in the bone marrow of cisplatin-treated rats, demonstrating the protective effect of melatonin administration.

Cisplatin is known to induce the formation of MNs in the bone marrow of rats. 53 We observed that 1 mg/kg of melatonin, administered concomitantly with cisplatin (group C), reduced the number of MNs when compared with the group treated with cisplatin only (group B).

Melatonin seems to protect hematopoietic cells; hence, it is possible that the negative effects of chemotherapy, including anemia and reduced cellular immunity, are minimized with the use of melatonin as an adjuvant.

## Conclusion

In the present study, the favorable findings regarding the prevention of cisplatin ototoxicity and genotoxicity following melatonin administration in rats, as well as its safety during treatment and beneficial effects on cancer prognosis, as previously demonstrated, suggest that melatonin may be a feasible option to reduce cisplatin toxicity.

The results observed highlight the applicability of melatonin as an adjuvant in the treatment of neoplasms. Additional studies on the use of melatonin as a complement to chemotherapy in humans, to avoid the undesirable side effects of cisplatin and improve patient survival, is an important goal for future research.
